# Hyperlipidemia Impaired Innate Immune Response to Periodontal Pathogen *Porphyromonas gingivalis* in Apolipoprotein E Knockout Mice

**DOI:** 10.1371/journal.pone.0071849

**Published:** 2013-08-16

**Authors:** Lang Lei, Houxuan Li, Fuhua Yan, Yin Xiao

**Affiliations:** 1 School and Hospital of Stomatology, Fujian Medical University, Fuzhou, China; 2 Institute and Hospital of Stomatology, Nanjing University Medical School, Nanjing, Jiangsu, China; 3 Bone Research Lab, Institute of Health and Biomedical Innovation, Queensland University of Technology, Brisbane, Australia; University of California Merced, United States of America

## Abstract

A finely-tuned innate immune response plays a pivotal role in protecting host against bacterial invasion during periodontal disease progression. Hyperlipidemia has been suggested to exacerbate periodontal health condition. However, the underlying mechanism has not been addressed. In the present study, we investigated the effect of hyperlipidemia on innate immune responses to periodontal pathogen *Porphyromonas gingivalis* infection. Apolipoprotein E-deficient and wild-type mice at the age of 20 weeks were used for the study. Peritoneal macrophages were isolated and subsequently used for the study of viable *P. gingivalis* infection. ApoE^−/−^ mice demonstrated inhibited iNOS production and impaired clearance of *P. gingivalis in vitro* and *in vivo*; furthermore, ApoE^−/−^ mice displayed disrupted cytokine production pattern in response to *P. gingivalis,* with a decreased production of tumor necrosis factor-α, interleukin-6 (IL-6), IL-1β and monocyte chemotactic protein-1. Microarray data demonstrated that Toll-like receptor (TLR) and NOD-like receptor (NLR) pathway were altered in ApoE^−/−^ mice macrophages; further analysis of pattern recognition receptors (PRRs) demonstrated that expression of triggering receptors on myeloid cells-1 (TREM-1), an amplifier of the TLR and NLR pathway, was decreased in ApoE^−/−^ mice macrophages, leading to decreased recruitment of NF-κB onto the promoters of the TNF-α and IL-6. Our data suggest that in ApoE^−/−^ mice hyperlipidemia disrupts the expression of PRRs, and cripples the host’s capability to generate sufficient innate immune response to *P. gingivalis*, which may facilitate immune evasion, subgingival colonization and establishment of *P. gingivalis* in the periodontal niche.

## Introduction

Periodontal disease,a common infection-driven chronic inflammatory disease, is characterized by destruction of the supporting tissues around the tooth. *Porphyromonas gingivalis*, a gram-negative oral anaerobe, is implicated as a predominant contributor to human periodontitis. Within the privileged anatomical niche of periodontal pocket, *P. gingivalis* can replicate and destroy periodontal tissue [1]. The innate immune system uses sentinel cells (monocytes, neutrophils, macrophages, dendritic cells), bearing pattern recognition receptors (PRRs), to recognize common molecular patterns on periodontal bacterial invaders, generating the immune and inflammatory responses, which leads to clearance of the bacteria [2].

Hyperlipidemia, resulting from impaired lipid metabolism by undue intake of cholesterol or genetic deficiency, has been suggested to exacerbate periodontal parameters in periodontitis patients in epidemiologic studies [3], [4], [5]. High-cholesterol food or fatty acid itself may depress immune function and bactericidal effect on *P. gingivalis* in the host [6], [7], and feeding animals a high-cholesterol diet impairs lipid metabolism and enhances periodontal destruction in lipopolysacchride (LPS) induced periodontitis in rats [8]. However, the underlying mechanism by which hyperlipidemia influences periodontal disease progress, especially the interaction between the innate immune system (including the PRRs) and the periodontal pathogen remains to be established.

Nearly half of the population above 20 years of age in the developed countries like USA and one third of the population above 35 years of age in the developing countries like China have hyperlipidemia [9], [10], whereas nearly half of the Chinese adults are inflicted with moderate to severe form of periodontal diseases [11]. Therefore, it is of great importance to understand the relationship between hyperlipidemia and periodontal disease. Although it has been demonstrated that periodontal diseases could influence lipid metabolism in the serum and the blood vessel [12], [13], it is not clear whether and how lipid metabolism anomaly in the blood may also affect periodontal disease progress.

Apolipoprotein E deficient (ApoE^−/−^) mice develop severe hyperlipidemia under normal diet condition [14]. In the present study, we tested our hypothesis that long term hyperlipidemia itself, rather than diet lipid, impaired the host immune response to periodontal infection in ApoE^−/−^ mice compared to healthy C57BL/6 mice. We found that long term hyperlipidemia impaired the immune response to *P. gingivalis* challenge by altering PRRs expression pattern in macrophages, leading an inhibited cytokine network response and decreased bacterial clearance; therefore, hyperlipidemia may lead to more severe periodontal bone loss.

## Materials and Methods

### Bacteria

*P*. *gingivalis* 33277 was obtained from the American Type Culture Collection, and cultured in brain heart infusion (BHI) broth (Oxoid,UK) supplemented with 5 µg/mL of hemin and 0.5 µg/mL of menadione at 37°C in an anaerobic environment consisting of 90% N_2_, 5% CO_2_ and 5% H_2_. Bacterial suspensions were prepared from cultures at their log phase of growth. Bacterial concentration was evaluated by measuring optical density at 600 nm (OD = 1, corresponding to 10^9^ bacteria/mL), and adjusted to the desired treatment concentration by dilution with phosphate buffered solution (PBS).

### Animals

One hundred six-week-old male C57BL/6 wild type (WT) mice and ApoE^−/−^ mice (Jackson Laboratories, BarHarbor, Me, US) were fed a regular mouse chow diet ad libitum until about 20 wks of age to experience long term hyperlipidemia. All animal studies were performed in accordance with the policies of the Institutional Animal Care and the study has been approved by the Animal Ethics Committee at Fujian Medical University.

### Serum Lipid Level

The serum total cholesterol (TC), triglyceride (TG), low density lipoprotein cholesterol (LDL-c) and high density lipoprotein cholesterol (HDL-c) concentration were determined by an enzymatic method using the protocols provided by the company (Biosino, Beijing, China). Detection sensitivity for TC, LDL-c, HDL-c and TG is 0.65 mmol/L, 0.50 mmol/L, 0.13 mmol/L and 0.15 mmol/L respectively.

### Peritoneal Infection

WT and ApoE^−/−^ mice were infected i.p. with *P. gingivalis* 33277 (5×10^7^ CFU). Peritoneal lavage was performed at indicated time post-infection. Serial 10-fold dilutions of peritoneal fluid were plated onto blood agar plates supplemented with hemin/menadione and cultured anaerobically for enumerating recovered peritoneal CFU. Furthermore, the periondontal lavage was centrifuged at 300×g at 4°C for 5 min. Then the cellular components were utilized in Western blot analysis for detecting iNOS protein, whereas the supernatants were used in both cytokine antibody array and ELISA to measure cytokine production.

### Cytokine Profile Analysis

Cytokine production in peritoneal fluid supernatants was analyzed for levels of 62 cytokines by Raybiotech Mouse Cytokine Antibody Microarray (AAM-CYT- G3, RayBiotech, Narcross, GA, US) according to the manufacturer’s instructions and as described earlier [15]. This antibody array detects simultaneously 62 mouse cytokines. Briefly, cytokine array membranes were blocked in 2 mL of 1×blocking buffer for 30 min, and then incubated with 1 mL of samples at room temperature for 2 h. Samples were then decanted from each container, and the membranes were washed three times with 2 mL of l×wash buffer I, followed by two washes with 2 mL of l×wash buffer II at room temperature with shaking. Membranes were then incubated in 1∶250 diluted biotin-conjugated primary antibodies at room temperature for 2 h and washed as described above before incubation in 1∶1000 diluted HRP-conjugated streptavidin. After incubation in HRP-conjugated streptavidin for 60 min, membranes were washed thoroughly and exposed to a peroxide substrate (detection buffers C and D) for 5 min in the dark before imaging. Membranes then were imaged using Axon GenePix laser scanner using cy3 channel. The signal intensities were imported into RayBio^®^ Analysis Tool. The densities were exported into Microsoft Excel, and the background intensity was subtracted prior to analysis. After subtracting background signals and normalization to positive controls, comparison of signal intensities for antigen-specific antibody spots between groups were utilized to determine relative differences in expression levels of each analyte (i.e., protein detected). Any ≥2-fold increase or ≤0.5-fold decrease in signal intensity for a single analyte between groups were considered a significant difference in expression, provided that both sets of signals were well above background (mean background±2 standard deviations).

### Macrophage Isolation and Cell Culture

Mouse macrophages were isolated from the peritoneal cavity upon thioglycolate-induced elicitation [16]. Harvested cells were subjected to centrifugation on a discontinuous Percoll gradient (55% and 30%; Sigma, US). Separated macrophages were resuspended in culture medium (RPMI 1640 supplemented with 10% fetal bovine serum), and cultured at 37°C under 5% CO_2_ atmosphere. The purity of macrophage preparations (>90%) was confirmed by flow cytometry using phycoerythrin-labeled anti-F4/80 (eBioscience, San Diego, CA, US).

### Macrophage Intracellular Bacteria Killing Assay

The intracellular *P. gingivalis* clearance capability of macrophages was determined by an antibiotic protection-based survival assay, as Wang et al described [17] **.** Briefly, following incubation of *P. gingivalis* with macrophages (at a multiplicity of infection [MOI] of 25∶1) for 1.5 h, extracellular nonadherent bacteria were removed by washing with PBS, whereas extracellular adherent bacteria were killed by addition of gentamicin (300 µg/mL) and metronidazole (200 µg/mL) for 1 h. After washing, macrophages remained to be cultured overnight. Internalized bacteria were released by lysis of macrophages in sterile distilled water for 20 min. Serial dilutions of the lysates were plated onto blood agar dishes supplemented with hemin and menadione, and cultured anaerobically for CFU enumeration.

### Phagocytosis

*P. gingivalis* was labeled with FITC as Hazenbos et al described [18], then uptake of FITC-labeled *P*. *gingivali* was detected by flow cytometry as Wang et al described [17]. Briefly, primary mouse macrophages were incubated at 37°C with FITC-labeled *P. gingivalis* at a MOI of 25∶1 for 30 min. Phagocytosis was stopped by cooling the incubation plates on ice. Cells were washed to remove nonadherent bacteria, then extracellular fluorescence (representing attached but not internalized bacteria) was quenched with 0.2% trypan blue in some groups. Both quenched and unquenched cells were analyzed by flow cytometry (% positive cells for FITC-P. *gingivalis* and mean fluorescence intensity [MFI]) using the FACSCalibur (Becton-Dickinson). Association (representing both adherence and phagocytosis) or phagocytic indices were calculated using the formula (% FITC-positive cells×MFI)/100. When macrophages were pretreated with cytochalasin D to block phagocytosis, macrophages that were stimulated with FITC-labeled *P. gingivalis* did not show significant fluorescence, which confirmed that trypan blue effectively quenched extracellular fluorescence.

### Cell Activation and Cytokine Assays

Mouse macrophages were stimulated with *P. gingivalis* at MOI of 25∶1. Cytokines released into culture supernatants were measured by ELISA according to the protocols of the manufacturers from the following sources: monocyte chemoattractant protein 1 (MCP-1), interleukin 1β (IL-1β), IL-6, and IL-12p70 (R&D System, Minneapolis, MN, US); tumor necrosis factor α (TNF-α), IL-10 (eBioscience, San Diego, CA, US).

### Microarray Analysis

Two hours after infection with P. gingivalis (MOI = 25∶1), total RNA of mouse macrophages was extracted using Trizol reagent (Invitrogen, Carlsbad, California, US) and was further purified by RNeasy micro kit and RNase-Free DNase Set (QIAGEN, Germany). Three independent experiments were performed for each condition. Total RNA was amplified, labeled and purified by using Affymetrix GeneChip 3′IVT Express Kit to obtain biotin labeled cRNA. Following array hybridization, slides (Affymetrix, Santa Clara, CA, US) were scanned by GeneChip Scanner 3000 and Command Console Software 3.1 (Affymetrix, US). Differentially expressed genes showing a statistical significance (p<0.05, one way analysis of variance) and ≥2-fold change between ApoE^−/−^ and the corresponding WT mice were further analyzed using SBC analysis system (Biochip, Shanghai,China): gene ontology (GO) analysis was applied to organize genes into hierarchical categories on the basis of cellular component, biological process and molecular function; enrichment pathway analysis (*Kegg* database) was implemented to uncover the major perturbed pathways. Complete microarray data have been deposited in the Gene Expression Omnibus public database (accession number GSE42061; http://www.ncbi.nlm.nih.gov/geo/query/acc.cgi?acc=GSE42061).

### Quantitative Real Time PCR (qPCR)

Total RNA was isolated from the cells using Trizol reagent. One microgram of total RNA was reverse-transcribed using Superscript II (Invitrogen, USA). Real-time quantitative PCR reactions were set up in triplicate using the SYBR® Premix Ex Taq™ II (Takara, Japan) and run on a 7900 HT Sequence Detection System (ABI, USA). All of the gene expression levels were normalized to the internal standard gene, *GAPDH*.

### Western Blot

The cellular components in peritoneal fluid were collected after centrifugation, then washed in Dulbecco’s phosphate-buffered saline (D-PBS) twice; for in vitro study, macrophages were harvested after trypsin-EDTA treatment, and rinsed with D-PBS for three times. Cytoplasmic protein was extracted using NE-PER nuclear and cytoplasmic extraction reagents and Halt protease inhibitor cocktails (ThermoScientific, Rockford, IL, US) according to the manufacturer’s protocol. Protein concentration was determined by a Bradford protein assay using BSA for standardization, and the cytoplasmic extracts were fractionated on 10% SDS–PAGE gels, transferred to a nitrocellulose membrane and incubated overnight with rabbit polyclonal iNOS primary antibodies (dilutions 1∶500) (Abcam, Cambridge, MA, US), followed by incubation with horseradish peroxidase (HRP)-conjugated goat anti-rabbit secondary antibody (1∶5, 000) (Genescript, Piscataway, NJ, US). A 1∶3,000 dilution of GAPDH rabbit polyclonal antibody (Genescript, USA) was used as a loading control. Bands were visualized by enhanced chemiluminescence (ECL) method**.** X-ray films were scanned and saved as grayscale JPEG files. The level of expression of proteins was analyzed by using the Image J software (National Institutes of Health, US), which can calculate area and pixel value statistics of user-defined selections. Each band was normalized to GAPDH. Each sample was tested in duplicate, and western blots were repeated three times.

### Flow Cytometry

Following blocking non-specific Fc receptor binding by anti-mouse CD16/32 antibody (eBioscience, US), mouse macrophages were incubated with phycoerythin (PE) labeled specific monoclonal antibodies (mAbs) or Ig isotype controls in PBS with 1% (w/v) BSA and 0.05% sodium azide (FACs buffer, eBioscience,US) on ice. Subsequently, the cells were washed, and analyzed by FACSCalibur(Becton-Dickinson). Mouse-specific mAbs to Toll-like receptors −2 (TLR-2) and TLR-4 were from eBioscience and mAb to triggering receptors expressed on myeloid cells-1 (TREM-1) was from R&D Systems.

### Chromatin Immunoprecipitation Assays

Mouse macrophages (1×10^7^cells per sample) were stimulated, washed with PBS, and fixed with 1% formaldehyde for 10 min at room temperature. Formaldehyde fixation was stopped with the addition of 1.25 M glycine. Sonicated chromatins were then immunoprecipitated (IP) with anti-p65 (Cell signaling Tech, US) or with control IgG Abs coupled to microbeads. DNAs from each sample (input or IP) were isolated by elusion, reverse cross-linking and proteinase K treatment. The DNA was then used as template to perform PCR amplification of the regions containing the proximal nuclear factor-κB (NF-κB) site in the promoters of TNF-α, IL-6 and IL-10.

### Statistical Analysis

Data were represented as the mean ± standard deviation (SD). Mean values between the groups were compared using analysis of variance (ANOVA). Differences were considered significant when *P*<0.05.

## Results

### Metabolic Characteristics of Experimental Animals

**Figure 1:**
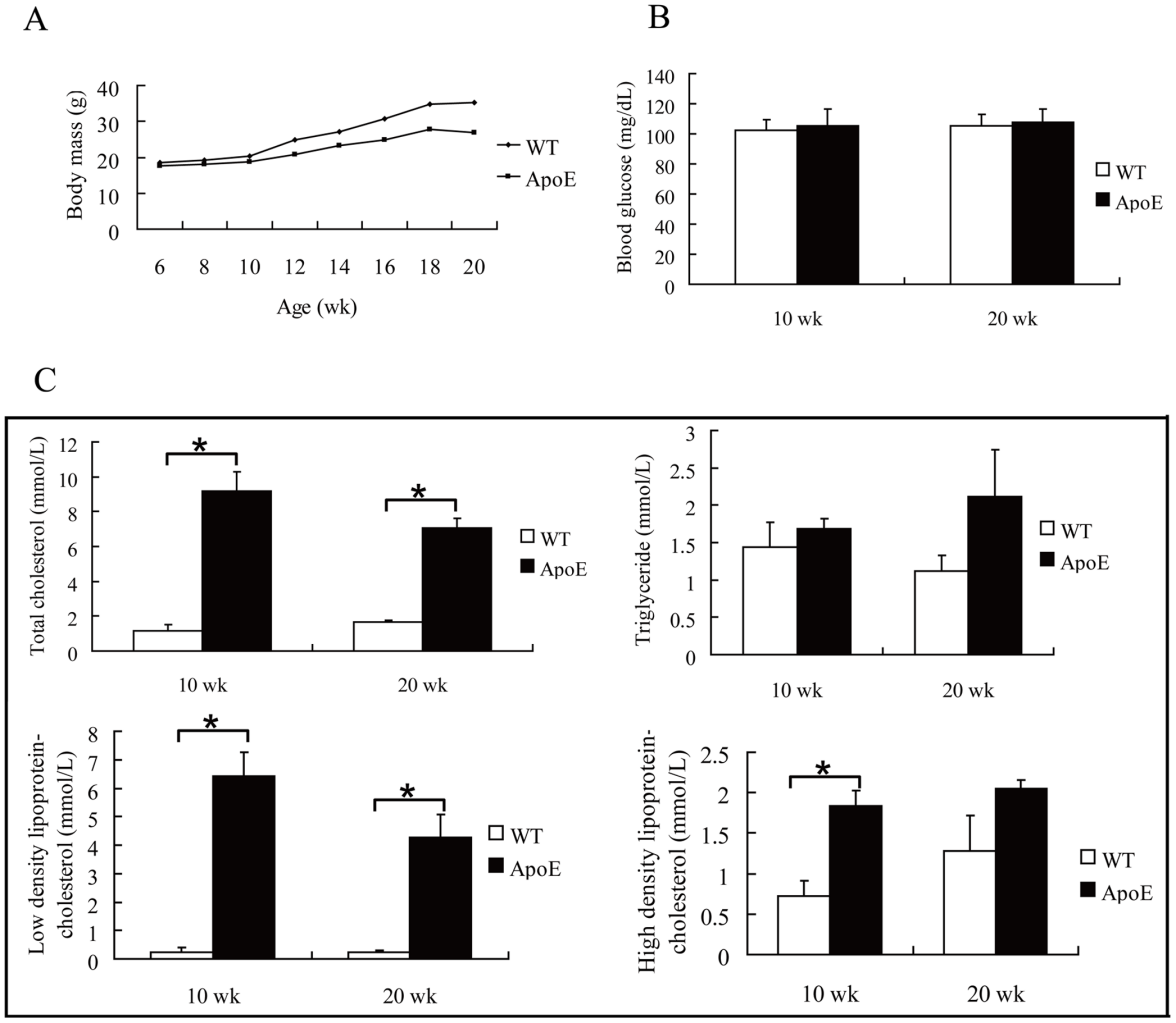
Metabolic characteristics of experimental animals. Six-week-old wild type (WT) and ApoE^−/−^ C57BL/6 mice were fed a normal chow diet for 14 weeks. Body mass (A), blood glucose (B) and serum lipid levels (C) were measured (n = 5). *, *P*<0.05.

ApoE^−/−^ mice weighed less than WT mice above 12 wks of age, and blood glucose level in ApoE^−/−^ mice was not significantly different from WT mice. ApoE^−/−^ mice showed significantly increased LDL-c levels in blood at 10 and 20 weeks, whereas serum triglyceride was not significantly different (Figure 1).

### Impaired Clearance of *P. gingivalis* in Hyperlipidemic Mice was Parallel with the Reduced iNOS Production

**Figure 2:**
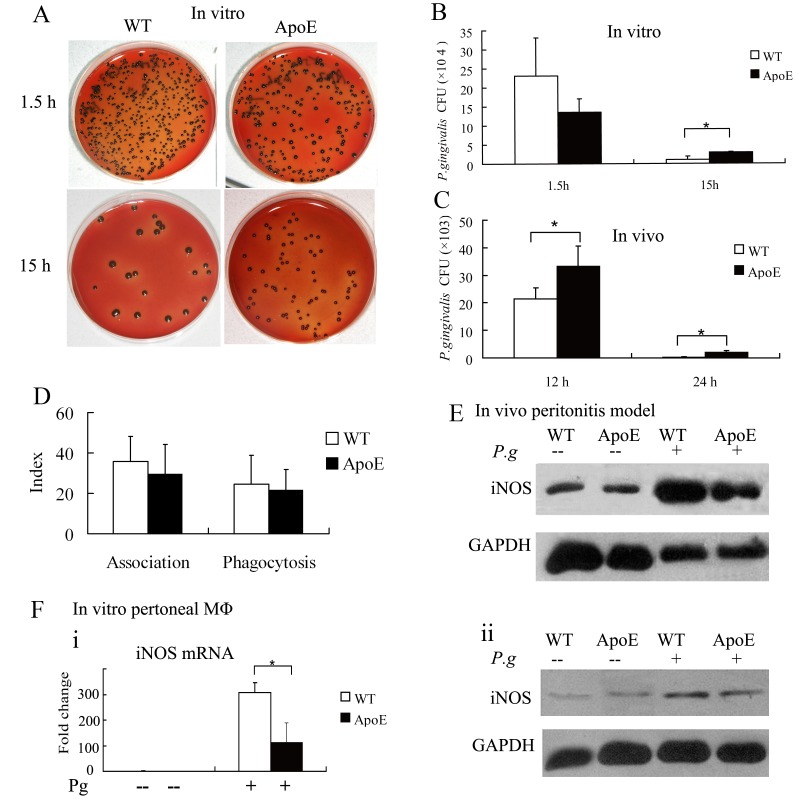
Impaired clearance of *P. gingivalis* by inhibiting iNOS induction in ApoE ^−/−^ mice. A and B, in vitro studies: peritoneal macrophages from wild type (WT) and ApoE ^−/−^ mice were infected with *P. gingivalis* (MOI = 25∶1) and CFU of internalized bacteria were determined at 1.5 and 15 h after infection. C, in vivo studies: mice were infected i.p. with *P. gingivalis* (5×10^7^). Serial dilutions of peritoneal fluid were plated for anaerobic growth and enumeration of recovered peritoneal CFU. D, peritoneal macrophages were incubated with FITC-labeled *P. gingivalis* (MOI = 25∶1) for 30 min. Association (i.e., representing both adherence and phagocytosis) or phagocytic indices were determined by flow cytometry, as described in Materials & Methods, using the following formula: (% positive cells for FITC-P. gingivalis×MFI)/100. E, 24 h after peritoneal infection, the induction of iNOS was determined by Western blot using the cellular components of the peritoneal fluid after centrifugation, data were normalized to GAPDH. F, mouse macrophages were infected with *P. gingivalis*, and iNOS production was determined by Western blot 24 h post infection (i), and iNOS mRNA was determined by qPCR 2 h post infection (ii). Results were means ±SD (*n* = 3) and were confirmed in repeated experiments. *, *P*<0.05.

Based on antibiotic protection-based intracellular killing assay, we found that macrophages from hyperlipidemic ApoE^−/−^ mice showed a decreased tendency to internalize *P. gingivalis* after 1.5 h incubation (*P*>0.05), whereas intracellular capability to remove *P. gingivalis* was impaired in macrophages from ApoE^−/−^ mice *in vitro* (figure 2A and 2B). Such impaired clearance in ApoE^−/−^ mice was confirmed in the peritonitis model *in vivo* (figure 2C).

In order to explore the changes in phagocytosis capability in macrophage, we utilized flow cytometry to measure the phagocytosis of FITC-labled *P. gingivalis*, and observed that macrophages from ApoE^−/−^ mice displayed similar binding and internalizing capability (figure 2D).

One of the fastest and most effective defense mechanisms for macrophages to clear invading bacteria is production of the free radical NO, which is mediated by the expression of inducible NO synthase (iNOS) [19]. Mice lacking iNOS demonstrated impaired killing of *P. gingivalis* and increased periodontal tissue damage upon *P. gingivalis* challenge [20], [21]. To investigate the cause of impaired clearance of *P. gingivalis* in ApoE^−/−^ mice, we analyzed the expression of iNOS. iNOS production was significantly decreased in the peritonitis model in ApoE^−/−^ mice when compared to WT mice (figure 2E). Macrophages from ApoE^−/−^ mice displayed severe disruption of iNOS expression at both the protein and mRNA levels after *P. gingivalis* infection (figure 2F-i, ii).

### Depressed Cytokine Production during *P. gingivalis* Infection in ApoE^−/−^ Mice

**Figure 3:**
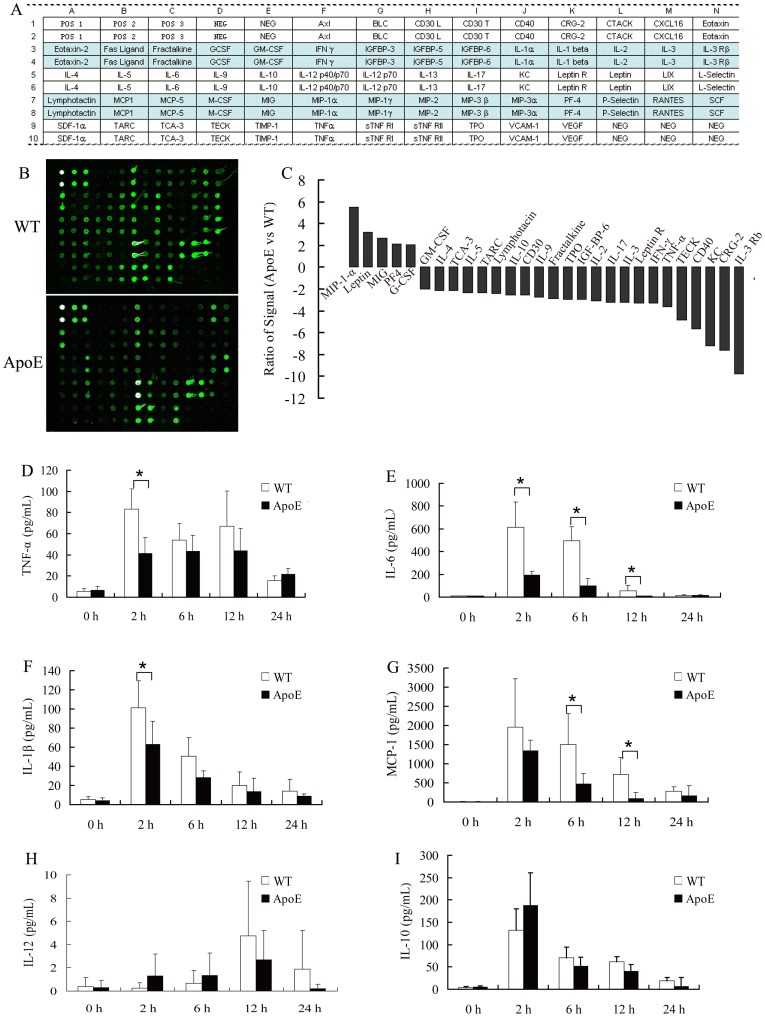
Inhibited cytokine production in ApoE^−/−^ mice in vivo. Wild type (WT) and ApoE^−/−^ mice were infected i.p. with *P. gingivalis* (5×10^7^). Peritoneal fluid of 6 h post infection was subjected to cytokine antibody array. Each cytokine is represented by duplicate spots (A). The cytokine array image represented results of three independent experiments (B). Cytokines with fold change >2.0 and *P*<0.05 was shown (C). TNF-α, IL-6, IL-1β, MCP-1, IL-12 and IL-10 levels at 0 h∼24 h was determined by ELISA (D-I) (mean ± SD; n = 3; representative of duplicate independent tests). *, *P*<0.05.

To further investigate the cause of impaired clearance of *P. gingivalis* in ApoE^−/−^ mice, we explored the cytokine profile during *P. gingivalis* infection. Six hours after peritoneal infection, we observed that 23 cytokines, including TNF-α, IL-2, IL-4 and interferon γ, were decreased in peritoneal fluid, whereas only 5 cytokines such as leptin, macrophage inflammatory protein-1α (MIP-1α) and platelet factor 4 (PF4) were increased in ApoE^−/−^ mice when compared to WT mice using mice cytokine arrays (figure 3A–C).

**Figure 4:**
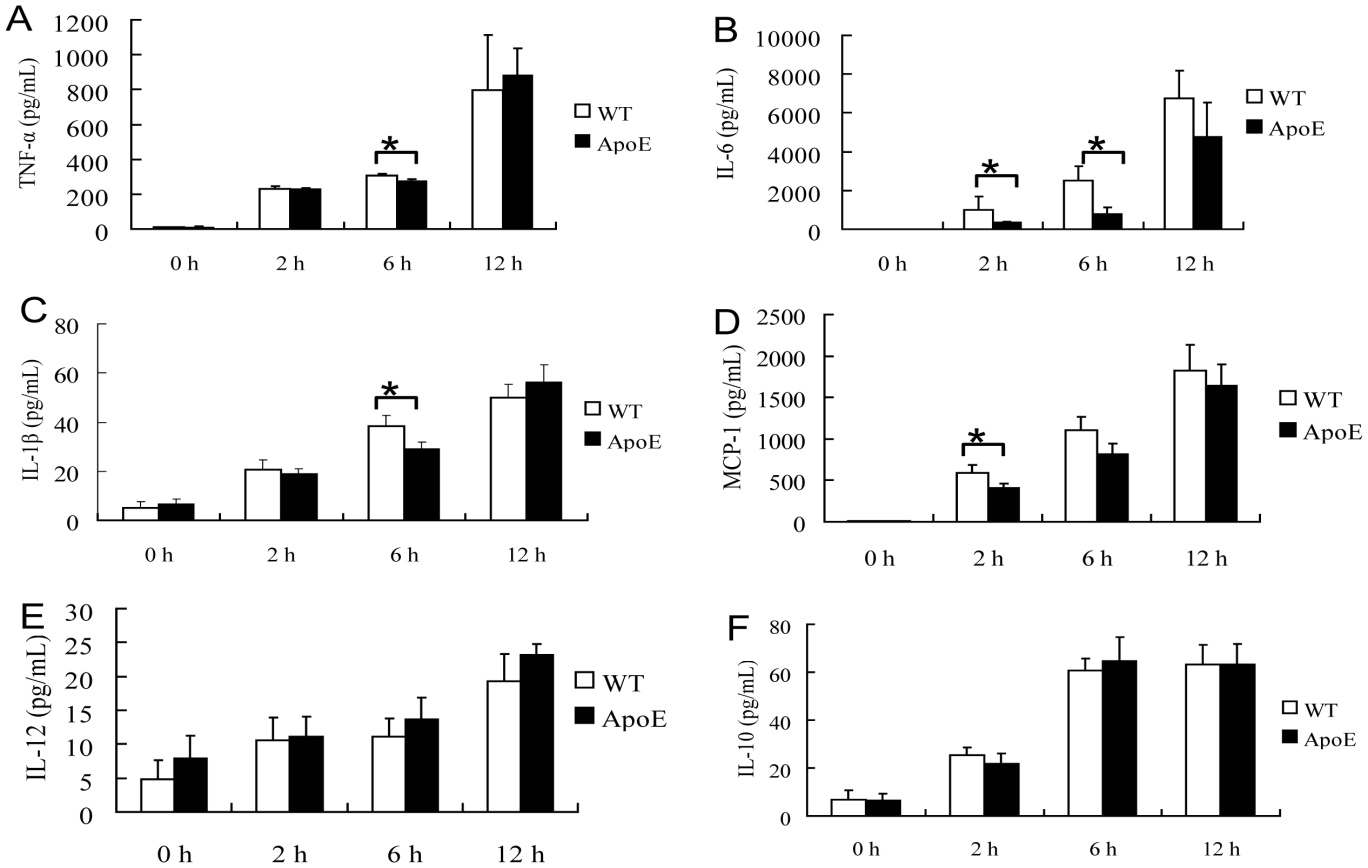
Inhibited cytokine production in ApoE^−/−^ mice macrophages. Macrophages from wild type (WT) and ApoE^−/−^ mice were exposed to live *P. gingivalis* (MOI = 25∶1). TNF-α, IL-6, IL-1β, MCP-1, IL-12 and IL-10 in culture supernatants were analyzed by ELISA (mean ± SD; n = 3). *, *P*<0.05.

Cytokine production pattern was also determined by ELISA at 0 h to 24 h after bacterial periotoneal infection. Significantly less induction of pro-inflammatory cytokines, TNF-α, IL-6, IL-1β and MCP-1, was found at various time post-infection in ApoE^−/−^ mice; whereas anti-inflammatory cytokine IL-10 was not affected, and IL-12 production was not detectable during infection (figure 3D–I). Similarly significantly blunt cytokine production was also found in *P. gingivalis* infected mice macrophages *in vitro* (figure 4A–F).

To further clarify whether the decreased cytokine production in ApoE^−/−^ mice was due to long term hyperlipidemia or the disruption of ApoE gene expression itself, we further analyzed cytokine production in ApoE^−/−^ and WT mice at the age of 6 wks in peritoneal infection model. In contrast to an impaired cytokine production pattern in ApoE^−/−^ mice at 20 wks of age, a modestly but significantly increased cytokine production pattern was found in peritoneal stimulation test in ApoE^−/−^ mice at 6 wks of age using ELISA (not depicted).

### Disrupted Gene Expression Profile after *P. gingivalis* Infection in ApoE^−/−^ Mice Macrophages

**Figure 5:**
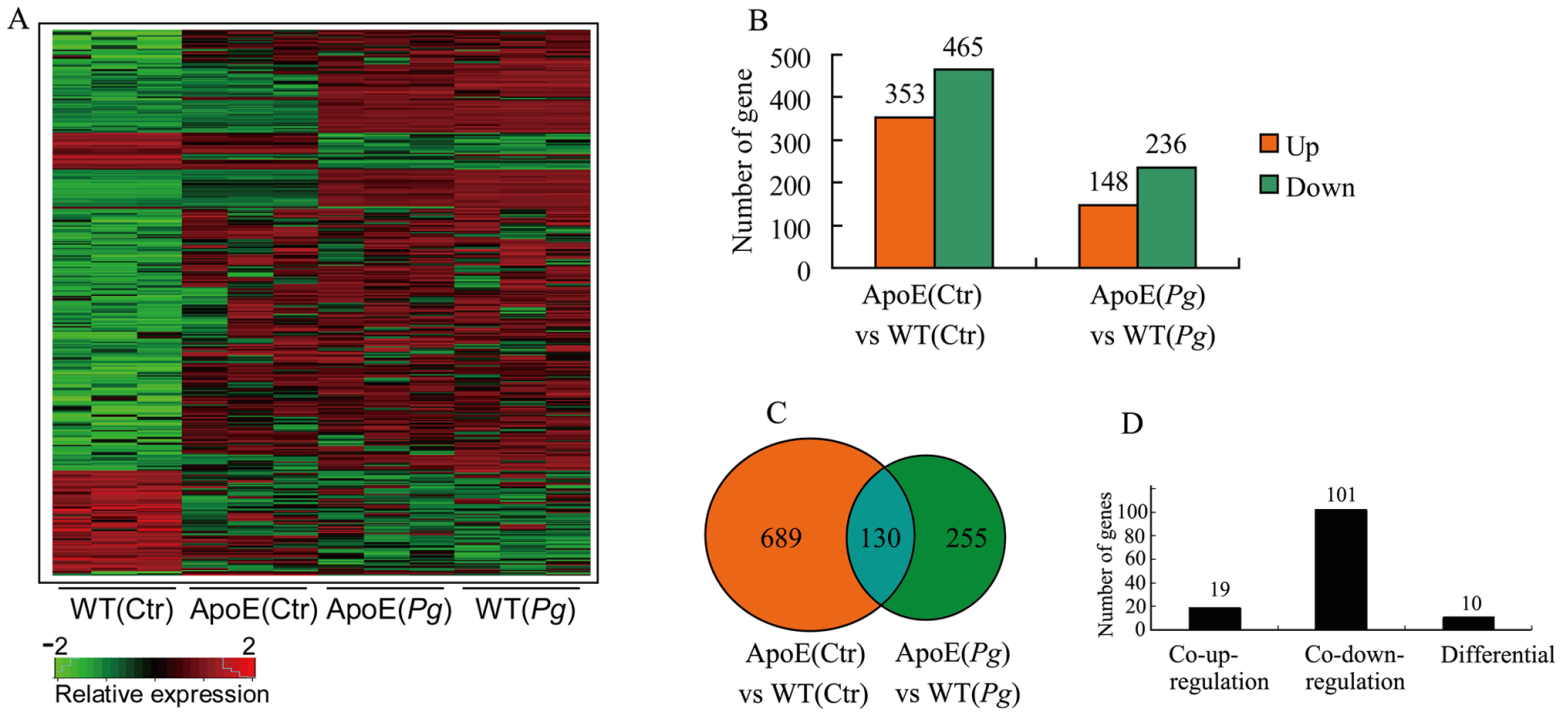
Disrupted gene profile in ApoE^−/−^ mice macrophages. Macrophages from wild type (WT) and ApoE^−/−^ mice were exposed to live *P. gingivalis* (MOI = 25∶1) for 2 h. Heat map of hierarchical clustering was utilized to reveal gene profiles; shown were qualified genes with fold change of >5.0 (P<0.05) between any groups (A). Red indicated up-regulation, whereas green indicated down-regulation, and black indicated no change. Differentially expressed genes in control and bacteria infected macrophages were categorized as up-regulated and down-regulated (B), then a Venn diagram was utilized to explore the logical relation between the difference expression genes(C), showing 130 genes were affected by hyperlipidemia in both the untreated and infected macrophages. Further analysis demonstrated 101 genes were down-regulated in the 130 genes (D).

A full genome microarray was utilized to investigate global gene transcription following *P. gingivalis* infection in macrophages, showing that mRNA transcription was inhibited in ApoE^−/−^ mouse macrophages. A hierarchical clustering algorithm was used to group qualifiers with similar patterns of expression. A heat map of genes of fold change of >5.0 was shown in figure 5A. 353 genes were up-regulated and 465 genes were down-regulated in ApoE^−/−^ control when compared to WT control; whereas 148 genes were up-regulated and 236 genes were down-regulated in *P. gingivalis* infected ApoE^−/−^ macrophages (figure 5B). We then further investigated the logical relations between the two comparisons, 130 genes were differentially expressed in both untreated and stimulated macrophages (figure 5C); furthermore, among such 130 genes, 101 genes were down-regulated and 19 were up-regulated in both untreated and stimulated macrophages (figure 5D).

To gain insights into the mechanism of disrupted macrophage response to live *P. gingivalis*, we focused on differential expression genes in *P. gingivalis* infected macrophages utilizing gene ontology and pathway analysis. We categorized these genes as down-regulated genes (236 genes) and up-regulated genes (148 genes). In *Biological Process* analysis, the up-regulated genes are involved in biological adhesion; whereas down-regulated genes take part in seven biological processes majorly involved in bacteria infection such as cell killing, immune system process and response to stimulus (Table 1). In enrichment pathway analysis (Kegg), the up-regulated genes are involved in extracellular matrix-receptor interaction and p53 signaling pathway; the down-regulated genes are involved in pathways such as cytokine and cytokine receptor interaction, NOD-like receptor (NLR) and Toll-like receptor (TLR) pathway (Table 2).

**Table 1:** Biological process affected by hyperlipidemia in *P. gingivalis* infected macrophages.

GOId	Name	Hits^1^	Percent^2^	Enrichment test *p*-value^3^
***Up-regulated***				
GO:0022610	biological adhesion	10	1.62%	0.0098
***Down-regulated***				
GO: 0008150	Cell killing	5	11.63%	2.00E-04
GO: 0002376	immune system process	41	5.07%	0
GO: 0050896	response to stimulus	53	2.39%	0
GO: 0051704	multi-organism process	8	2.68%	1.15E-02
GO: 0048518	positive regulation of biological process	21	1.66%	0.0325
GO:0048518	negative regulation of biological process	19	1.65%	4.34E-02

1 Total count of difference expression genes.

2 The proportion of total count versus the number of genes in the biological process.

3 Only the biological process with p-value <0.05 were shown.

Only biological processes that are related to innate immune response to bacterial infection were shown**.**

**Table 2:** The list of pathway category (Kegg) in difference expression genes in *P. gingivalis* infected macrophages.

Kegg Pathway Name	Hits	Percent	Enrichmenttest *p*-value
***Up-regulated***			
ECM-receptor interaction	3	3.57%	7.0E-4
p53 signaling pathway	2	2.63%	0.0105
***Down-regulated***			
Antigen processing and presentation	3	2.88%	0.0047
Chemokine signaling pathway	15	7.39%	0.0
Cytokine-cytokine receptor interaction	23	8.49%	0.0
Cytosolic DNA-sensing pathway	5	8.62%	0.0
ECM-receptor interaction	2	2.38%	0.0297
Endocytosis	4	1.67%	0.0074
JAK-STAT signaling pathway	10	6.25%	0.0
Natural killer cell mediated cytoxicity	7	4.35%	0.0
NOD-like receptor signaling pathway	5	7.69%	0.0
p53 signaling pathway	2	2.63%	0.0248
RIG-I-like receptor signaling pathway	6	8.75%	0.0
T cell receptor signaling pathway	3	2.27%	0.0088
TGF-beta signaling pathway	4	4.35%	2.0E-04
Toll-like receptor signaling pathway	8	7.69%	0.0

Only pathways correlated to innate response to bacterial infection were shown.

### Gene Regulation was Confirmed by qPCR

The microarray results pointed to an inhibited gene response to viable *P. gingivalis*. In follow-on studies, we selected genes that are potentially implicated in inflammatory response to *P. gingivalis*: *interleukin-1 receptor-associated kinase 1 binding protein 1 (Irak1bp1), signal transducer and activator of transcription 1 (Stat1), Tnfa, IL6, IL12a, MCP1.* qPCR results confirmed gene microarray efficiency. For example, *Irak1bp1,* an inhibitor in TLR pathway, was down-regulated by 1.68 fold in microarray analysis and 1.86 fold in qPCR in untreated macrophages, and by 39.89 and 55.98 fold respectively in *P. gingivalis* infected macrophages (Table 3).

**Table 3:** qPCR validation for selected genes affected by hyperlipidemia.

Gene symbol	Full name	ApoE(blank)vs C57(blank)		ApoE(*Pg*)vs C57(*Pg*)	
		Microarray	qPCR	Microarray	qPCR
*Stat1*	signal transducer and activatorof transcription 1	−3.03	−2.99	−2.08	−1.69
*Irak1bp1*	interleukin-1 receptor-associatedkinase 1 binding protein 1	−1.68	−1.86	−39.89	−55.98
*Tnfa*	tumor necrosis factor α	2.29	−2.18	−1.25	−1.23
*Il6*	interleukin 6	1.07	1.08	−2.14	−3.78
*Il12a*	interleukin 12A	−2.08	−2.45	−3.09	−2.11
*Mcp1*	monocyte chemotactic protein-1	−3.09	−1.88	−2.08	−3.04

### Different Pattern Recognition Receptors (PRRs) Expression Pattern in ApoE^−/−^ Mice

**Figure 6:**
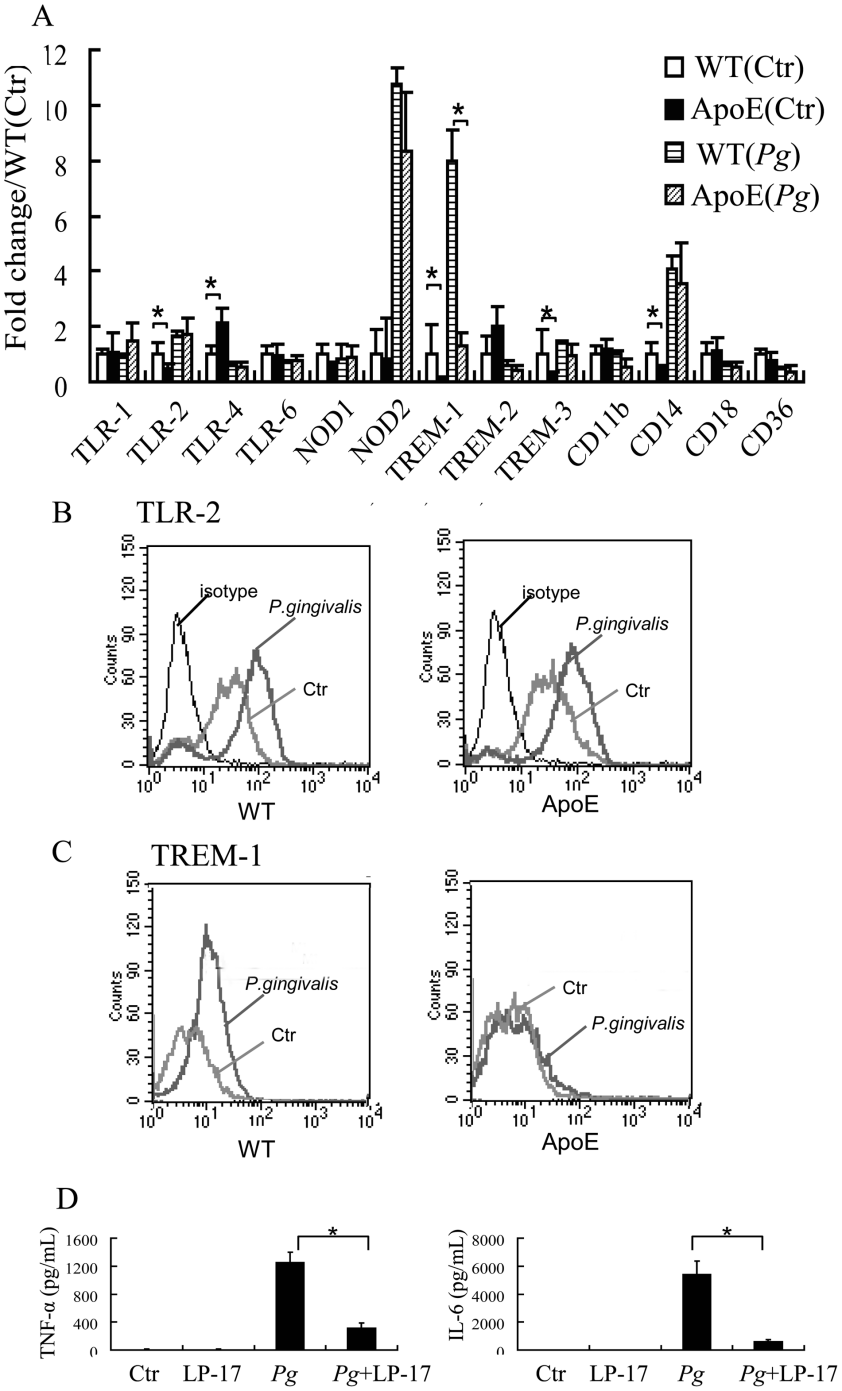
Different pattern recognition receptors (PRRs) expression between wild type (WT) and ApoE^−/−^ mice macrophages. Mice peritoneal macrophages were infected with *P. gingivalis* (MOI = 25∶1) for 2 h, then mRNA of PRRs related to TLR and NLR pathway were determined by qPCR (A). TLR-2 and TREM-1 expression were confirmed by flow cytometry (B and C), showing significantly inhibited expression of TREM-1 in ApoE^−/−^ mice macrophages. Blockage of TREM-1 with LP-17 peptide pretreatment decreased TNF-α and IL-6 production significantly at 6 h after *P. gingivali*s infection in peritoneal macrophages. (n = 3). *, *P*<0.05.

Following the clue that TLR and NLR pathway were affected *in* ApoE^−/−^ mice, we further analyzed the PRRs expression. As shown in figure 6A, transcription of *TLR-2*, *-4*, *triggering receptors expressed on myelod cells -1* (*TREM-1*), *TREM-3* and *CD14* was decreased in untreated macrophages of ApoE^−/−^ mice, whereas only TREM-1 mRNA was significantly decreased in *P. gingivalis* infected macrophages in ApoE^−/−^ mice. TLR-2 protein expression was not affected in both the untreated and stimulated macrophages, while TREM-1 expression was significantly decreased in ApoE^−/−^ mice after bacteria stimulation (figure 6B). To test the effect of TREM-1 inhibition on immune response, we blocked TREM-1 with LP-17 peptide 2 h before stimulation with *P. gingivalis,* and TREM-1 blockage significantly inhibited TNF-α and IL-6 production upon *P. gingivalis* infection at 6 h in WT mice macrophages (figure 6C). Furthermore, macrophages from WT mice treated by LP-17 peptide displayed less production of cytokine when compared to macrophages from ApoE^−/−^ mice.

### Less p65 Binding to Gene Promoters after Decreased TREM-1 Expression

**Figure 7:**
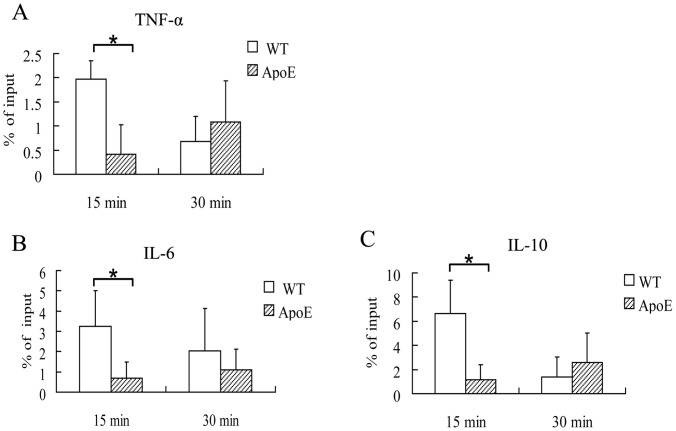
Inhibited recruitment of NF-κB to cytokine promoters in ApoE^−/−^ mice macrophages. Peritoneal macrophages from wild type (WT) and ApoE^−/−^ mice were treated with live *P. gingivalis* (MOI = 100∶1) for 15 min and 30 min. NF-κB p65 polyclonal antibody precipitated DNA was quantified by real time PCR, and the results were expressed as percentage of ChiP DNA to input DNA. *, P<0.05. (n = 3).

As TREM-1 is an amplifier of both the TLR and NLR pathway, down-regulation of TREM-1 may lead to inadequate signal transduction upon *P. gingivalis* infection. To further characterize its effect on the innate immune response to bacteria invasion, we focused on the transcriptional factor NF-κB, which plays a key role in regulating the immune response to infection. We further evaluated the capability of NF-κB p65 subunit’s binding ability to DNA at TNF-α, IL-6 promoter (classic pro-inflammatory locus) and IL-10 promoter (anti-inflammatory locus). Recruitment of p65 to all 3 promoters in macrophages from ApoE^−/−^ mice at 15 min was decreased in comparison with WT mice (Figure 7). Although no difference was observed in the recruitment of NF-κB to gene promoters at 30 min between ApoE^−/−^ mice and WT mice macrophages, binding to κB sites in ApoE^−/−^ mice macrophages at 30 min was still less than that in WT mice macrophages at 15 min.

## Discussion

In normal mice, a well-orchestrated cytokine network maintains a finely-tuned response to bacterial challenge. Cytokines such as TNF-α and IL-1β that are considered harmful to periodontal tissue in chronic inflammation play important roles in the control of pathogen infection in the acute inflammation [22]. The principal finding of the present study is that hyperlipidemia, a kind of metabolic anomaly accompanying many obesity subjects, disrupts host capability to clear periodontal pathogen *P. gingivalis*, as a result of impaired capability to mount sufficient immune response to *P. gingivalis.* To our knowledge, this is the first report that systematically investigated the potential mechanisms by which hyperlipidemia interferes with host’s normal innate response to periodontal bacterial infection.

Hyperlipidemia may directly influence disease induction and indirectly modulate immune responses [23]. Lipoproteins can bind LPS and decrease LPS-stimulated cytokine production [24]; therefore, the inhibited inflammatory response in our study may partly be attributed to the elevated low density lipoprotein in the circulation system. In the present study, we found that the ApoE^−/−^ mice failed to generate a robust immune response. Congruent with our results, similar observation was also made during *Klebsiella pneumoniae* and *Leishmania major* infection [23], [25]. As ApoE itself has an immunoinhibitory effect on inflammation [26], animals deficient in ApoE at an early age exhibit increased cytokine production in response to *Listeria monocytogenes* [27], *Mycobacterium tuberculosis* [28],*Klebsiella pneumoniae* [29], and display inhibited type I inflammatory response to *E. coli* LPS, a classical TLR4 agonist, in vivo [30].

In contrast to the impaired cytokine production in ApoE^−/−^ mice at 20 wks of age, a slightly increased cytokine release to *P. gingivalis* was observed *in vivo* in ApoE^−/−^ mice at 6 wks of age. Therefore, the inhibited immune response in ApoE^−/−^ mice at 20 wks of age can be attributed to long term hyperlipidemia, which overcomes the effects of ApoE deficiency, leading to decreased cytokine release and iNOS production. Furthermore, iNOS deficiency may impair host’s ability to kill *P. gingivalis* and aggravate soft tissue damage and alveolar bone loss [20], [21], [31]. Our study is congruent with the study of Shamshiev et al [32], showing that dyslipidemia inhibits TLR induced activation of CD8α^-^ dendritic cells in ApoE^−/−^ mice in aged mice (20–30 wks-age), rather than young mice (5 wk).

In agreement with the inhibited cytokine production in ApoE^−/−^ mice, microarray data showed that overall gene response in ApoE^−/−^ mice was inhibited, and biological processes related to innate immune response such as *cell killing*, *immune system process*, *response to stimulus*, became impaired. Such gene expression profile would alter the clearance of *P. gingivalis* in macrophages *in vitro* and *in vivo*. To further discover the mechanism of hyperlipidemia interfering with the innate response, pathway analysis was conducted, showing that TLR and NLR pathway was influenced by hyperlipidemia.

Recognition of invasive pathogens by immune cells relies on their capacity to detect microbial- or pathogen-associated molecular patterns (PAMPs) [19]. This process involves the coordinated actions of different membrane-associated or intracellular pattern-recognition molecules, including TLRs and NLRs [33]. Ligand-activated receptors trigger the mitogen-activated protein kinases (MAPKs), NF-κB, and interferon-related factor (IRF) signal transduction pathways that induce the transcription and production of immune genes, including cytokines that are critical for the activation of innate immunity [34].

Following the clue in microarray results, we further demonstrated that although TLR-1, -2, -4 and -6, NOD-1 and -2 expressions upon *P. gingivalis* challenge was not affected, whereas TREM-1 became significantly inhibited in ApoE^−/−^ mice. In addition, in the present study, blockage of TREM-1 by LP-17 peptide significantly inhibited IL-6 and TNF-α production in response to *P. gingivalis* in macrophages. As TREM-1 is an amplifier of both the TLR and NLR signal transduction pathway [35], [36], an inhibited TREM-1 expression may be a potential cause leading to decreased inflammatory signal transduction and cytokine release upon *P. gingivalis* infection. Such effect was confirmed by the decreased recruitment of NF-κB onto the promoters of the pro-inflammatory TNF-α and IL-6, and the anti-inflammatory IL-10. Although we observed an inhibited TREM-1 expression in ApoE^−/−^ mice macrophages, caution is still required for the result explanation. Over-expression of TREM-1 in ApoE^−/−^ macrophages would further shed insight into the contributory role of TREM-1 in the disrupted cytokine production in ApoE^−/−^ mice.

Understanding kinetic properties of NF-κB interaction with its target genes, i.e. the binding and unbinding, may help us understand the inflammatory response to *P. gingivalis.* After degradation of their cytoplasmic inhibitors (IκBs), NF-κB dimers translocate to the nucleus and bind to thousands of 9–10 nt κB sites dispersed in the genome [37], [38], generating complexes with a half-life of 45 min [39]. As promoter-bound NF-κB is in dynamic equilibrium with nucleoplasmic dimers, promoter occupancy and transcriptional activity oscillate synchronously with nucleoplasmic NF-κB [40]. In addition to its ability to bind to NF-κB in cytosol, IκBα can also dissociate NF-κB from DNA and export it to the cytosol [41]. In our experiment, we observed that binding of NF-kB in ApoE^−/−^ mice macrophages at 30 min was similar to that in WT mice at 30 min, but it was still less than that in WT mice at 15 min. Therefore, NF-κB in ApoE^−/−^ mice macrophages demonstrated a later and less competent binding to its promoters, leading to the decreased production of IL-1β, IL-6, TNF-α, MCP-1 both in vivo and in vitro.

Activation of monocytes by TLRs stimulants, such as LPS, results in the rapid production of proinflammatory cytokines, including TNF-α, IL-1β, and IL-6, followed later by the secretion of the anti-inflammatory IL-10. However, in our study, IL-10 production in ApoE^−/−^ mice was not altered in vivo and in vitro, which was inconsistent with the decreased recruitment of NF-κB to IL-10 gene promoters. As both NF-κB and MAPK signal pathways, including ERK, JNK, and p38, are involved in the classical LPS-induced IL-10 expression [42], [43], further exploration on MAPK pathway might unravel the possible role of NF-κB binding to the IL-10 promoter.

Although we observed an inhibited innate immune response and TREM-1 expression in hyperlipidemic ApoE^−/−^ mice, the possible mechanism by which hyperlipidemia influences PRRs has not been fully understood. Macrophages express a host of receptors, such as CD36 and scavenger receptor A, which bind and internalize modified forms of LDL. Thus serum lipid may be incorporated into cell membranes and cytoplasm, altering immune response to PAMPs. Indeed, patients with hyperlipidemia abnormities displayed altered lipid composition of the cell membrane and an increased cholesterol contents in cytoplasm in erythrocytes and lymphocytes [44], [45], [46], [47]. Furthermore, macrophages from ApoE^−/−^ mice showed 3-fold higher membrane cholesterol compared with wild-type macrophages [48].

Subtle changes in the lipid composition of the plasma membrane, particularly the cholesterol content, may result in the modification on lipid rafts, which provide spatial organization for transmembrane proteins and act as signaling platforms responsible for coordinating outside-in and inside-out signal transduction [49]. Toll-like receptors (TLR) and interleukin receptors are found within the lipid rafts of cells treated with pro-inflammatory LPS [50], [51], [52]. Therefore, alteration in lipid contents in circulation system may influence cholesterol composition in cell membrane and affect lipid rafts dynamics, leading to altered inflammatory response to bacteria infection.

*P. gingivilis* may exploit various receptors in cell membrane to facilitate its invasion, such as TLR2, CD14, CXC-chemokine receptor 4 (CXCR4), complement receptor 3 (CD11b/CD18) [17], [53], [54], [55]. In our study, macrophages from ApoE^−/−^ mice showed inhibited transcription of TLR2, TREM-1, TREM-3, and CD14 in quiescent state, whereas only TREM-1 expression was influenced by hyperlipidemia in bacteria stimulated macrophages.

In addition to membrane receptors, *P. gingivalis* utilizes lipid rafts as signaling and entry platforms [56]. *P. gingivalis* colocalizes with lipid rafts in a cholesterol-dependent way, depletion of cellular cholesterol using methyl-beta-cyclodextrin resulted in about 50% inhibition of *P. gingivalis* uptake, and this effect was reversed by cholesterol reconstitution [56]. Macrophages from ApoE^−/−^ mice show higher membrane cholesterol than wild-type macrophages [48]. Therefore, an increased internalization might be expected in macrophages from ApoE^−/−^ mice. In contrast, we failed to observe changes in phagocytosis in macrophages. Possible roles of TREM-1 in the inside-out mechanism of *P. gingivalis* need to be explored further.

Consistent with Bostanci et al [57], we observed that TREM-1 takes part in the immune response to *P. gingivalis* in mice macrophages. Following stimulation by LPS in vitro and in vivo, monocytes secrete a soluble form of TREM-1 (sTREM-1) [58]. sTREM-1 has been found to correlate with inflammatory disease, such as sepsis [59], [60], relapsing polychondritis [61] and inflammatory bowel diseases [62]. Elevated oral and systemic levels of soluble TREM-1 has been found in periodontitis [63], [64]. Therefore, monitoring changes in sTREM-1 level would help understand inflammation and immune response in the periodontal niche.

**Figure 8:**
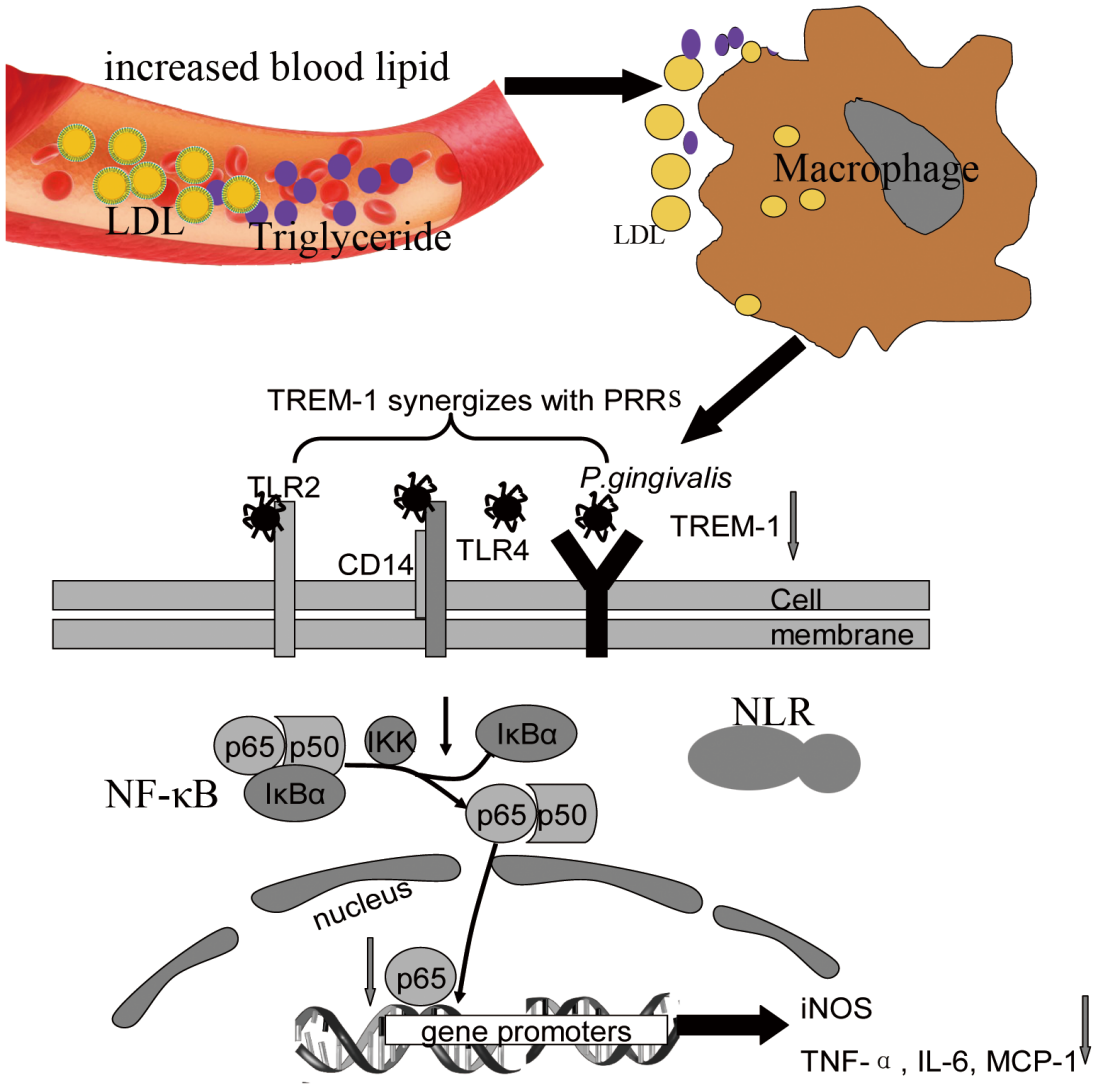
Proposed mechanism for impaired host innate immune response to viable *P. gingivalis* infection by hyperlipidemia in ApoE^−/−^ mice. Increased blood lipid would be incorporated into cell membrane and intracellular components of macrophages, leading to the decreased TREM-1 expression upon *P. gingivalis* infection. Reduced TREM-1 activation resulted in inadequate signal transduction, less p65 nuclear translocation and binding to gene promoters region at DNA; therefore, innate immune responses including iNOS production and release of cytokines, such as TNF-α, IL-6 and MCP-1, were disrupted in the hyperlipidemic host. LDL, low density lipoprotein; TREM-1, triggering receptors expressed on myeloid cells-1; PRRs, pattern recognition receptors, TLR, Toll-like receptors; NLR, NOD-like receptors; NF-κB, nuclear factor κB; IκB, inhibitory κB; IKK, IκB kinase complex; iNOS, inducible nitric oxide synthase; IL-6, interleukin 6; MCP-1, monocyte chemoattractant protein 1; TNF-α, tumor necrosis factor α.

The possible mechanism by which hyperlipidemia impairs innate immune responses to viable *P. gingivalis* infection has been illustrated and summarized in figure 8. Briefly, increased serum lipid would alter the cholesterol component in the cell membrane and cytoplasm of macrophages, which might further affect PRR expression on the cell membrane upon *P. gingivalis* challenge. Disrupted activation of TREM-1, a kind of PRRs amplifying the TLR transduction pathway, could lead to inhibited p65 nuclear translocation and binding to gene promoters region. Consequently, innate immune responses including iNOS production and cytokines release, such as TNF-α, IL-6 and MCP-1, might be suppressed in the hyperlipidemic host.

Given that chronic periodontitis is characterized by alternate periods of exacerbation and quiescence [65], the impaired TREM-1 expression and the inhibited innate immune response would have a significant influence on the periodontal disease progress. This inability to mount a sufficient inflammatory response may lead to inadequate elimination of the bacteria, which would in turn grow and proliferate in the periodontal niche, and cause more severe periodontal damage.

In conclusion, we have shown that hyperlipidemia inhibits inflammatory response in macrophages, which in turn cripples host’s capability to remove the bacterial infection. These findings suggest that hyperlipidemia can predispose the host to oral infection, by impairing proper immune response to bacteria challenge.
